# Spontaneous Intracranial Hypotension Presenting With Frontotemporal Dementia: A Case Report

**DOI:** 10.3389/fneur.2018.00673

**Published:** 2018-08-17

**Authors:** Ahmet Ozyigit, Costas Michaelides, Konstantinos Natsiopoulos

**Affiliations:** ^1^University of Nicosia Medical School, Nicosia, Cyprus; ^2^Department of Neurology, American Medical Center, Nicosia, Cyprus; ^3^Department of Neurology, University of Nicosia Medical School, Nicosia, Cyprus; ^4^Department of Radiology, American Medical Center, Nicosia, Cyprus

**Keywords:** intracranial hypotension, cerebrospinal fluid, dementia, dysarthria, narcolepsy

## Abstract

Spontaneous intracranial hypotension (SIH) is a rare and often underdiagnosed condition, which commonly results from a cerebrospinal fluid leak. The classic clinical presentation of SIH is a postural headache and dizziness. Less frequent complications include nausea, neck stiffness, and even coma. This case report describes a 70-year-old woman with an initial complaint of postural headaches and sleep attacks, who developed a 22-month progressive history of personality and behavioral changes, cognitive decline, urinary incontinence, chorea, and dysarthria. Although no specific cerebrospinal fluid leak was identified, the patient was suspected of having SIH and her symptoms completely reversed after a 2-month course of steroids. This case highlights that SIH represents a rare and reversible cause of a wide spectrum of neurological symptoms, including dementia. Neurologists should be aware of this diagnosis when evaluating patients with neurological signs and symptoms that cannot otherwise be explained.

## Introduction

Spontaneous intracranial hypotension (SIH) is a rare condition, with an estimated incidence of 5 in 100,000 (1). The typical features of SIH include a throbbing postural headache that occurs or becomes worse in the upright position, dizziness, diffuse pachymeningeal enhancement and a “brain sag” visualized by magnetic resonance imaging (MRI), and a low cerebrospinal fluid (CSF) opening pressure during a lumbar puncture (2). As CSF volume decreases, compensatory vasocongestion and interstitial edema of the dura mater maintain a dynamic equilibrium (3). Although these classic features help diagnose most patients with SIH, there has been an increasing number of patients who either do not meet the classic diagnostic criteria or who present with uncommon manifestations such as taste alterations (4), limb paresthesias (5), behavioral changes (6), and Parkinsonian-like symptoms (7).

One of the main causes of SIH is a CSF leak, which usually occurs in the spine, causing hypoliquorrhea (8). However, clinical evidence suggests that patients suffering from SIH often have a normal opening pressure (9, 10). Although the clinical symptoms used to diagnose SIH may vary, downward displacement of the brain due to the loss of CSF buoyancy, often called “brain sag,” is very specific to SIH (4). The degree of displacement may help explain the wide spectrum of symptoms associated with SIH, ranging from occasional postural headaches to coma (11).

Rare complications of SIH include reversible frontotemporal dementia, Parkinsonian-like symptoms, quadriplegia, coma, and even death (12–15). An increasing number of patients diagnosed with SIH have accompanying behavioral changes diagnosed as frontotemporal dementia, spawning the term “frontotemporal brain sagging syndrome” (15). In this report, we describe an unusual presentation of SIH in a patient who presented with progressive cognitive deficits, increased somnolence, dysarthria, chorea, and urinary incontinence. To our knowledge, this is the first report of SIH associated with this constellation of symptoms in the literature.

## Case report

A 70-year-old female retired healthcare professional initially presented with a 10-day history of severe headaches without associated symptoms and with no history of head trauma. The patient had similar headaches in the past, but of shorter duration and lower intensity. The headaches developed suddenly and would occur when she leaned forward or stood up from a recumbent position. She described the headaches as an intense pressure-like sensation. Over-the-counter analgesics were ineffective. A few weeks after the onset of headaches, the patient started experiencing sleep attacks, wherein she would fall asleep during a conversation and even while standing up. She also reported having very realistic dreams that she often confused with reality. A multiple sleep latency test (MSLT) was normal, which refuted the clinical suspicion of narcolepsy. The sleep study did show mild sleep apnea, and she was prescribed continuous positive airway pressure (CPAP). Although the headaches spontaneously resolved within a few weeks of their onset, her sleep symptoms did not improve despite using CPAP for 6 months. She also lacked energy and motivation to participate in daily and social activities.

**Figure 1:**
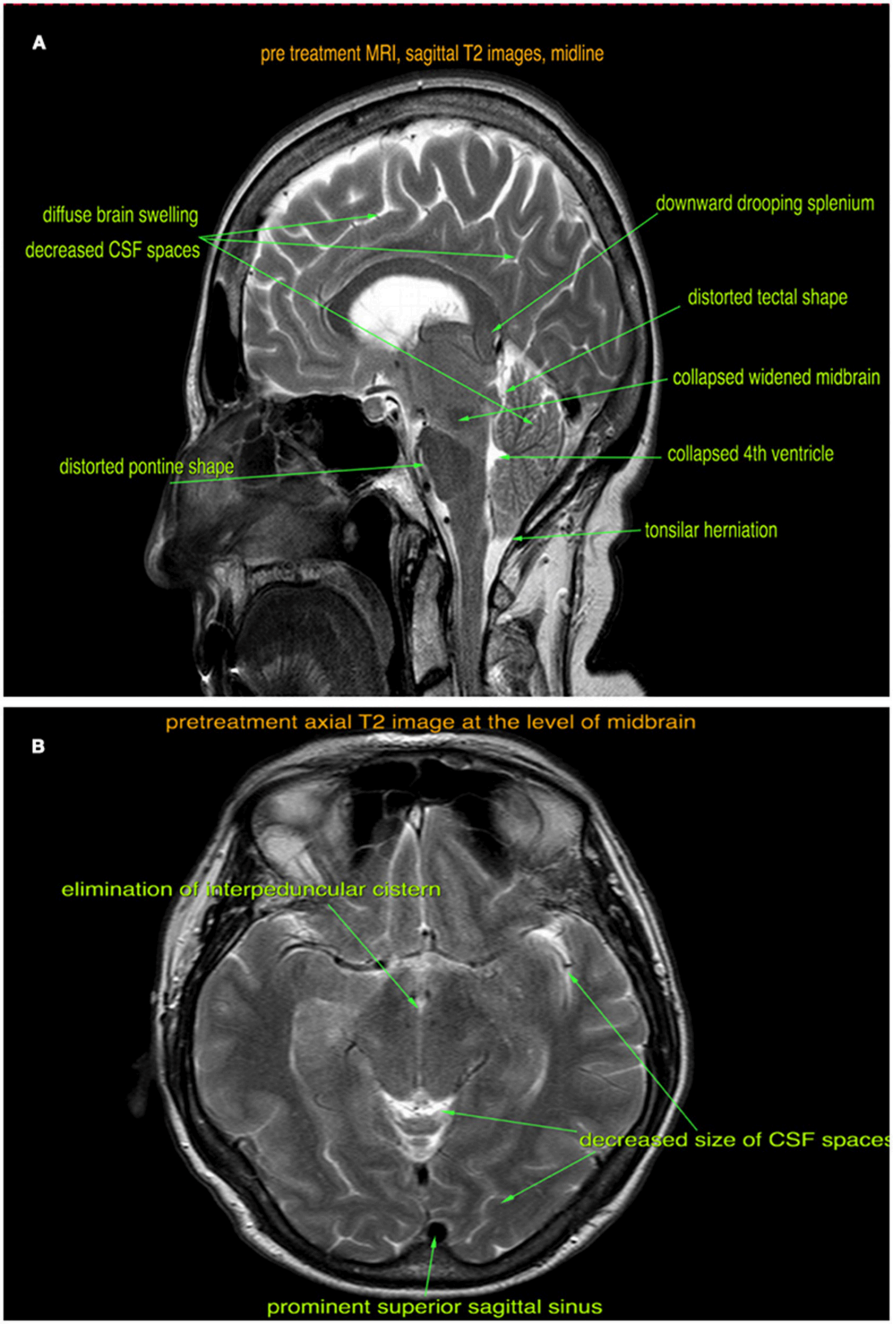
Pre-treatment sagittal **(A)** and axial **(B)** T2-weighted MRI images show brain sagging, tonsillar herniation, diffuse brain swelling, and venous sinus congestion.

Over the next 6 months, she developed severe dysarthria, although she lacked insight into her speech pathology. She was referred to a neurologist for further testing. Her EEG was normal and her brain MRI (Figure 1) showed mild frontotemporal atrophy. Based on these findings, she was diagnosed with frontotemporal dementia (FTD).

About 1 year after the onset of symptoms, the patient received a second opinion from another neurologist and underwent another brain MRI scan. This time, she received a clinical diagnosis of narcolepsy and was started on 100 mg modafinil tablets. The patient was also seen by a psychiatrist, who prescribed donepezil for her mild cognitive impairment. Her speech returned to normal, her sleep attacks completely resolved, and she felt more energetic. Unfortunately, all of her symptoms except for the sleep attacks reappeared 3 days after starting this treatment. The dose of modafinil was increased to 200 mg after 1 week, which improved her daytime sleep attacks, but did not improve her level of energy or motivation. About 1 month later, she started complaining of urinary incontinence, which was more severe than the stress incontinence she previously experienced.

Over the next 3 months, the patient developed additional symptoms. She became less concerned with her personal hygiene and developed impulsive and inappropriate behaviors such as eating fruit in the supermarket without paying and using her hands to eat. She also developed memory problems, although her family reported that these were not severe. She also developed involuntary choreatic movements, of which she was unaware, such as eye blinking, stretching her neck during a conversation, mouth movements, and tapping her fingers (not a tremor). With the emergence of these new symptoms, the opinion of a third neurologist was sought, who ordered an immunological panel, another EEG, another brain MRI scan, and a neuropsychological assessment. Based on the results of these studies, the patient was diagnosed with a behavioral variant of FTD. She continued taking modafinil for her sleep attacks and was prescribed a selective serotonin reuptake inhibitor for her behavioral symptoms.

The patient was seen at our institution at the peak of her symptoms, about 18 months after the onset. By this time, she was also experiencing occasional fecal incontinence. She was well kempt, fully alert, with expressive difficulties and dysarthria. Her Mini-Mental State Exam was 20/30 with deficits in orientation to time, short-term memory, calculation, repetition, and copying. She had mild spasticity of the right upper extremity during passive movements, but the rest of her neurological exam was unremarkable. A retrospective review of the brain MRI scans (Figures 1A,B) revealed diffuse pachymeningeal gadolinium enhancement and sagging of the brainstem, which were consistent with intracranial hypotension. An MRI scan of the whole spine was also performed, which showed 30 perineural cysts at all levels of the spine, but no evidence of a CSF leak. An epidural blood patch was considered; however, both the patient and her family wanted to try conservative treatment with a tapered course of steroids.

Based on a previous case report of a patient with a similar constellation of symptoms, we prescribed 1,000 mg of intravenous methylprednisolone daily for 3 days, followed by 80 mg of oral prednisone daily, which was decreased by 10 mg every week. For the last month of treatment, she received 5 mg of oral prednisone daily.

**Figure 2:**
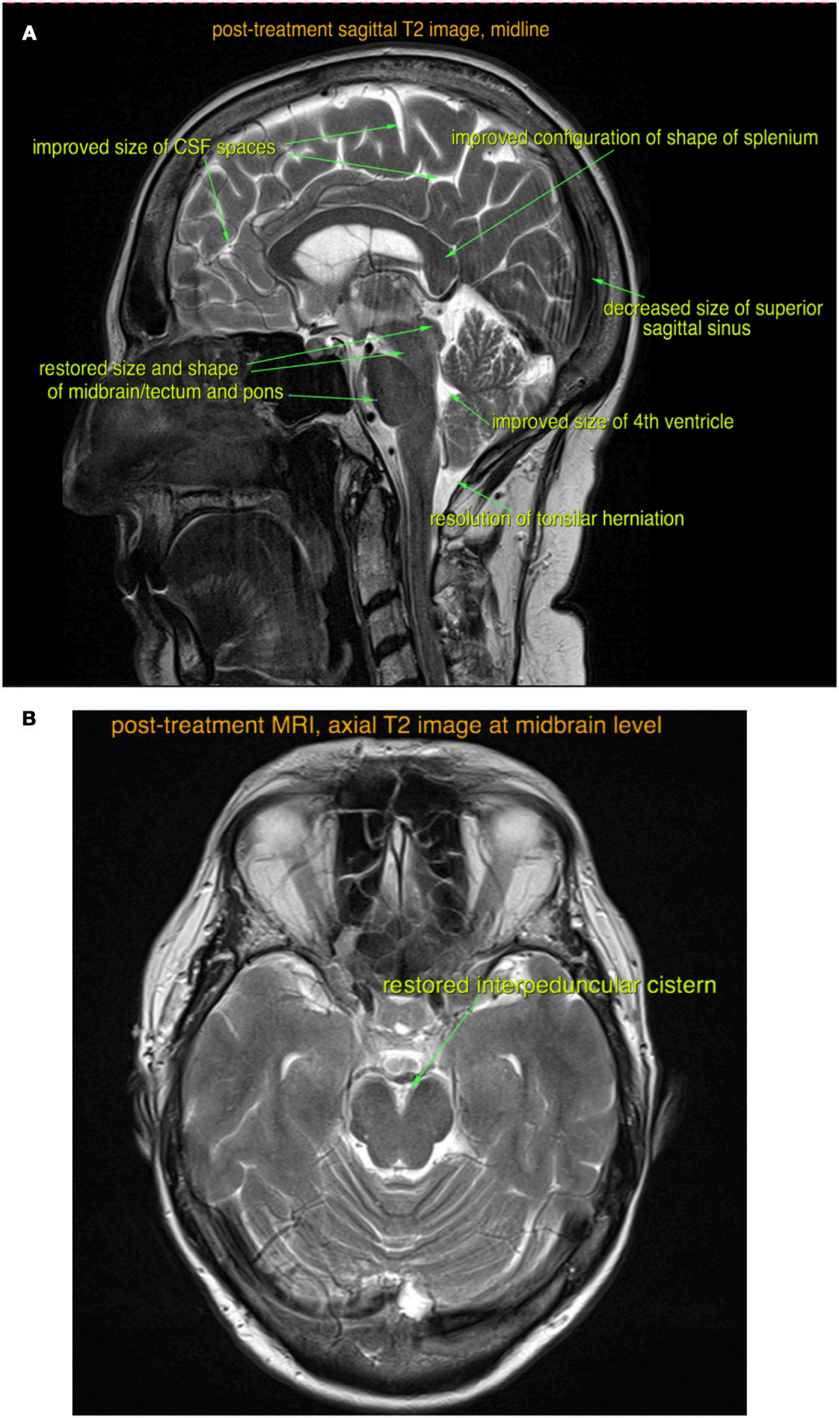
Post-treatment sagittal **(A)** and axial **(B)** T2-weighted MRI images show almost complete resolution of the findings of intracranial hypotension.

Four weeks after starting steroid treatment, her cognitive dysfunction and speech difficulties started to improve. Six weeks into treatment, she was able to perform activities of daily living such as cooking and washing dishes. After 8 weeks of treatment, her urinary and fecal incontinence fully resolved and she started to feel more energetic. A repeat brain MRI scan was performed at the end of the fourth month (Figures 2A,B), which showed reduced brain swelling, less venous sinus congestion, and resolution of the tonsillar herniation.

## Discussion

Although headaches and stupor are frequently reported symptoms of SIH, the patient in this case not fit the classic presentation of SIH. A literature review revealed no prior cases or diagnostic guidelines for this constellation of symptoms. Some case reports reported FTD-like symptoms in patients with SIH (11, 13–15). However, the reported symptoms in these case studies were not as wide-ranging as the symptoms in our case, nor was there any mention of sleep attacks or speech-related problems in any of the previous reports. Another report described a patient with brain sagging syndrome who presented with choreoathetosis, which may have been more severe than our patient's chorea (16). In addition, our patient improved more slowly than patients in previously reported cases; therefore, it is possible that she had the condition for a longer duration. It is possible that she had this condition for a very long time before it started manifesting as distinct symptoms. We cannot, however, make such an assumption based on what evidence we have. This is possibly one of the main limitations of this report.

In conclusion, the clinical manifestations of SIH are not yet fully understood, and SIH should be included in the differential diagnosis of patients who present with a constellation of diverse neurological symptoms that cannot otherwise be explained.

## Consent

The patient provided written informed consent for the publication of this manuscript.

## Author contributions

AO wrote the first draft of this manuscript. CM and KN helped revise the manuscript and substantially contributed to the clinical interpretation of this patient's findings.

### Conflict of interest statement

The authors declare that the research was conducted in the absence of any commercial or financial relationships that could be construed as a potential conflict of interest.

## References

[1] Couch JR. Spontaneous intracranial hypotension: the syndrome and its complications. Curr Treat Options Neurol. (2008) 10:3–11. 10.1007/s11940-008-0001-5 18325294

[2] Balkan II Albayram S Ozaras R Yilmaz MH Ozbayrak M Mete B. Spontaneous intracranial hypotension syndrome may mimic aseptic meningitis. Scand J Infect Dis. (2012) 44:481–8. 10.3109/00365548.2012.664776 22404365

[3] Antony J Hacking C Jeffree RL. Pachymeningeal enhancement-a comprehensive review of literature. Neurosurg Rev. (2015) 38:649–59. 10.1007/s10143-015-0646-y 26264063

[4] Schievink WI. Misdiagnosis of spontaneous intracranial hypotension. Arch Neurol. (2003) 60:1713–8. 10.1001/archneur.60.12.1713 14676045

[5] Pakiam AS Lee C Lang AE. Intracranial hypotension with parkinsonism, ataxia, and bulbar weakness. Arch Neurol. (1999) 56:869–72. 10.1001/archneur.56.7.869 10404990

[6] Peng PW. Intracranial hypotension with severe neurological symptoms resolved by epidural blood patch. Can J Neurol Sci. (2004) 31:569–71. 10.1017/S0317167100003838 15595269

[7] Tian W Zhang J Chen J Liu Y Chen X Wang N. A quantitative study of intracranial hypotensive syndrome by magnetic resonance. Clin Neurol Neurosurg. (2016) 141:71–76. 10.1016/j.clineuro.2015.12.014 26745515

[8] Spontaneousintracranial hypotension resulting in stupor caused by diencephalic compression. Neurology (1998) 50:1854–7. 9633740 10.1212/wnl.50.6.1854

[9] Kranz PG Tanpitukpongse TP Choudhury KR Amrhein TJ Gray L. How common is normal cerebrospinal fluid pressure in spontaneous intracranial hypotension? Cephalalgia (2016) 36:1209–17. 10.1177/0333102415623071 26682575

[10] Schievink WI. Spontaneous spinal cerebrospinal fluid leaks and intracranial hypotension. JAMA (2006) 295:2286–96. 10.1001/jama.295.19.2286 16705110

[11] Goto S Ohshima T Yamamoto T Shimato S Nishizawa T Kato K. Successful steroid treatment of coma induced by severe spontaneous intracranial hypotension. Nagoya J Med Sci. (2016) 78:229–36. 27303109 PMC4885822

[12] National Organization for Rare Disorders Spontaneous intracranial hypotension. Rare Disease Information (2017). Available online at: https://rarediseases.org/rare-diseases/spontaneous-intracranial-hypotension/

[13] Agarwal P Menon S Shah R Singhal BS. Spontaneous intracranial hypotension: two cases including one treated with epidural blood patch. Ann Indian Acad Neurol. (2009) 12:179–82. 10.4103/0972-2327.56318 20174499 PMC2824935

[14] Samson K Hypotension may cause frontotemporal dementia. Neurol Today (2002) 2:35–6. 10.1097/00132985-200209000-00013

[15] Wicklund MR Mokri B Drubach DA Boeve BF Parisi JE Josephs KA. Frontotemporal brain sagging syndrome: an SIH-like presentation mimicking FTD. Neurology (2011) 76:1377–82. 10.1212/WNL.0b013e3182166e42 21502595 PMC3087405

[16] Mulroy E Caldwell J Anderson NE Snow B. Brain sagging syndrome presenting with chorea. Neurol Clin Pract. (2017) 7:407–8. 10.1212/CPJ.0000000000000347 29620082 PMC5874458

